# Albumin Nanoparticles as Multifunctional Carriers for Advanced Therapeutics

**DOI:** 10.3390/pharmaceutics18010130

**Published:** 2026-01-20

**Authors:** Bogusława Konopska, Janusz Sokołowski, Anna Woźniak, Mikołaj Kondracki, Jakub Federowicz, Wojciech Grodzki, Agnieszka Bronowicka-Szydełko, Katarzyna Madziarska

**Affiliations:** 1 Department of Laboratory Diagnostics, Faculty of Pharmacy, Wroclaw Medical University, 50-556 Wroclaw, Poland; 2 Department of Emergency Medicine, Faculty of Medicine, Wroclaw Medical University, 50-556 Wroclaw, Poland; janusz.sokolowski@umw.edu.pl; 3 Clinical Department of Diabetology, Hypertension and Internal Diseases, Faculty of Medicine, Wroclaw Medical University, 50-556 Wroclaw, Poland; anna.wozniak@umw.edu.pl (A.W.); katarzyna.madziarska@umw.edu.pl (K.M.); 4 Faculty of Medicine, Wroclaw Medical University, 50-367 Wroclaw, Poland; mikolaj.kondracki@student.umw.edu.pl (M.K.);; 5 Department of Biochemistry and Immunochemistry, Faculty of Medicine, Wroclaw Medical University, 50-369 Wroclaw, Poland; agnieszka.bronowicka-szydelko@umw.edu.pl

**Keywords:** albumin, drug delivery systems, nanoparticles, theranostics

## Abstract

Modern medicine requires effective, continuous, and safe therapies, which largely depend on the targeted delivery and activity of the drug. This goal can be achieved by designing drug delivery systems with improved pharmacokinetic properties and enhanced drug transport to the affected tissue. Human serum albumin (HSA) is an attractive carrier for the synthesis of therapeutic nanoparticles, several of which have already been approved by the United States Food and Drug Administration (FDA). The success of Abraxane as an effective treatment for metastatic breast cancer and non-small cell lung carcinoma, the application of Optison in ultrasound imaging, and the use of Nanocoll as an agent for SPECT diagnostics in sentinel node localisation confirm the strong potential of albumin-based systems. Further benefits are expected in patients with soft tissue cancers, as LadRx is seeking FDA marketing approval for Aldoxorubicin. The future of oncology lies in theranostics, which combines a tumour-localising factor on one platform with a drug targeting cancer cells and a factor that activates the cytotoxicity of the drug after it reaches the target tissue. This article presents recent advancements in albumin-based nanoparticles for drug delivery, targeting, and imaging. It also briefly discusses methods of synthesis and surface modification of albumin nanocarriers to enable targeted delivery to pathological sites. Finally, it outlines the latest approaches in multimodal theranostic platforms, highlighting albumin’s potential to improve cancer therapy.

## 1. Introduction

**Figure 1:**
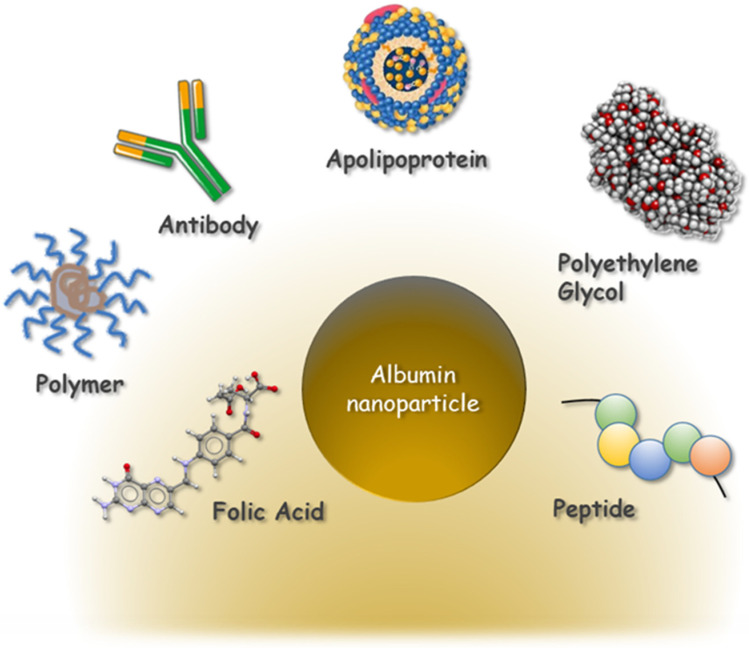
Surface modification strategies of albumin nanoparticles (NPs) for different delivery approaches.

Albumins are widely used in the synthesis of nanocarriers for medical applications. Their popularity arises from their broad availability, low production cost, biodegradability, and non-immunogenicity [1,2]. Albumin-based drug delivery nanosystems combine the intrinsic advantages of albumin as a carrier with the unique benefits of nanoparticles. They are characterised by favourable pharmacokinetics, high drug delivery efficiency, and low cytotoxicity. Additional important features include the facile incorporation of therapeutic agents, high loading capacity, controlled release capability, and presence of functional groups that allow simple surface modification [3,4]. These properties enable the covalent conjugation of drugs to albumin nanoparticles, thereby extending their circulation half-life and promoting tissue-specific targeting. Albumin-based delivery systems exist in a variety of sizes and morphologies, the most common being nanoparticles, microspheres, albumin-coated liposomes, albumin microbubbles, and nanocapsules [5,6,7]. The main strategies for surface modification of albumin nanoparticles are summarised in Figure 1.

- Folic acid: enhanced cellular uptake and increased water solubility [8];
- Polymer: penetration across the blood–brain barrier and tumour targeting [9];
- Monoclonal antibody: improved drug targeting [10];
- Apolipoprotein: drug transport and uptake into the brain [11];
- Polyethylene glycol (PEG): extended systemic circulation and enhanced intratumoural accumulation [12];
- Peptide: targeted tissue uptake and drug delivery [13,14].

Due to their high therapeutic efficiency and low toxicity, albumin nanostructures have a wide range of biomedical applications [15]. The most advanced research on albumin nanostructures is focused on anticancer therapy, which in conventional approaches is associated with systemic side effects on healthy tissues, progressive drug resistance, and, in particular, the need for frequent drug administration. Encapsulating chemotherapeutics in albumin nanoparticles has often reduced toxic effects while simultaneously improving therapeutic outcomes [16,17]. Furthermore, the natural tendency of albumin to accumulate in cancer cells has been exploited to develop new diagnostic tools for in vivo imaging of tumour tissue [18,19]. FDA approval and commercialisation of several albumin-based formulations have stimulated further research in nanopharmacology and nanodiagnostics for cancer therapy [20,21,22,23,24].

The commercial success of albumin-based nanocarriers began in 1995 with the approval of Nanocoll^®^, [99mTc]-labelled nanocolloidal human albumin, for lymphoscintigraphy, primarily to identify sentinel lymph nodes (SLNs) in breast cancer/melanoma and to assess lymphatic system integrity. But the real milestone in albumin-based formulation was nab technology, developed and patented by American Bioscience. The nab technology enabled encapsulation of poorly water-soluble therapeutics into nanoparticles, avoiding the use of solvents often associated with a high risk of side effects. The first drug manufactured using nab technology and approved by the FDA for clinical use was nab-paclitaxel. Abraxane^®^ is a 130 nm polymeric nanoparticle based on HSA and paclitaxel, which exists in a non-crystalline, amorphous form. After intravenous administration, the nanoparticles immediately dissociate into soluble 10 nm cytostatic-albumin complexes. Avoiding organic solvents in the formulation eliminates the need for premedication with steroids and antihistamines and allows also shortening the infusion time of a higher single dose (260 mg/m^2^) than Taxol (175 mg/m^2^) [25]. The nab-paclitaxel is used in the treatment of metastatic breast cancer in adult patients who have failed first-line treatment and who cannot receive standard anthracycline therapy. The preparation is also registered for combination therapy with gemcitabine as first-line treatment in metastatic pancreatic adenocarcinoma and non-small cell lung cancer in combination with the cancer medicine carboplatin when the patient cannot have surgery or radiotherapy. Additionally, nab-paclitaxel is being investigated for use in the treatment of metastatic melanoma, bladder cancer, colorectal cancer, and small bowel cancer. More recently, new nab technology-based formulations (nab-sirolimus) have been shown to be powerful therapeutics for oncology and rheumatology [26]. Fyarro^®^ was approved for the treatment of advanced malignant perivascular epithelial cell tumours (PEComa) in 2021. In addition, an albumin-binding nanobody directed against human TNF-α was approved in Japan for the treatment of rheumatoid arthritis in 2022 under the trade name Nanozora^®^ [27]. Other albumin-based drug formulations are currently at different clinical stages and are comprehensively presented in the literature [28,29].

Although albumin nanoformulations are particularly explored for chemotherapeutics, several fields of medicine can benefit from the unique properties of albumin [30]. For example, crosslinked albumin formulations are successfully applied as a surgical sealant, promoting both wound care and healing [31]. Encapsulation of antibacterial or antifungal agents in an albumin shell can improve the safety and efficacy of therapy [32]. The albumin receptor gp60, abundantly expressed in the blood–brain barrier, is being studied to enhance the intracerebral transport of antiepileptic drugs and drugs used in neurodegenerative diseases [33]. The versatility of albumin drug carrier stems from the ability to control drug delivery and release at the pathological site, and the role of albumin in this process is briefly discussed below.

## 2. Properties of Albumin Nanoparticles in Drug Targeting

Albumin as a drug carrier was originally acknowledged to overcome the lack of specificity and selectivity of conventional chemotherapeutics because of the observed accumulation of the albumin-bound drug in the tumour microenvironment [5]. Enhanced uptake of albumin nanoparticles by the tumour is driven by two mechanisms: passive transport and active transport. Passive transport is primarily associated with the EPR effect (enhanced vascular permeability and retention), explained through the specific morphological and metabolic features of malignant or inflamed tissue. Rapid and chaotic angiogenesis in such pathological conditions leads to a leaky capillary network, combined with defective lymphatic drainage, resulting in the easy penetration of macromolecules through the impaired endothelial layer and their retention in the abnormal tissue. As a result, anticancer drugs linked to albumin selectively accumulate in tumour tissue, increasing therapeutic efficacy while limiting adverse effects on healthy cells [34,35]. Moreover, cancer cells internalise albumin molecules efficiently because albumin serves as a primary source of energy and building materials for proliferation. Studies have shown that hypoalbuminemia observed in cancer is mainly due to intensified catabolism of this protein by tumour cells [1]. Albumin accumulation is facilitated by the increased expression of SPARC (secreted protein, acidic and rich in cysteine), also known as osteonectin, characteristic of solid tumours. This protein exhibits significant homology with the endothelial cell surface receptor gp60 (albondin), a known receptor for albumin. Overexpression of osteonectin in cancer cells promotes high invasiveness and metastasis while enhancing albumin uptake and the effectiveness of cytostatics encapsulated in albumin nanoparticles [36]. The significance of the EPR effect in drug delivery to tumour tissues, although evident in mouse models with transplanted human cancer cells, remains inconsistent in clinical practice due to the heterogeneous nature across tumour types and patients. This unsatisfactory therapeutic outcome caused novel approaches to improve the EPR effect by additional strategies influencing the microenvironment of surrounding tissues. These strategies include improving tumour perfusion, increasing vascular permeability for nanoparticles, or reducing the density of stromal cells [37]. At the molecular level, the approaches utilise tumour microenvironment (TME)-specific molecular markers, tumour-specific pathophysiological conditions, and TME-specific enzymes. The combined strategies to improve the EPR effect aim at physical alteration of TME by photodynamic, sonodynamic, and radiation therapies. Additionally, some approaches remodel the tumour vasculature using anti-angiogenic agents, affecting permeation and disruption, and enlarge the endothelial pores by using vasodilators and vasoactive cytokines [38].

However, nanosystems for drug delivery can be specifically modified with ligands to target tumour sites selectively. This last feature is defined as active transport. Active transport relies on delivering drugs to cancer cells via receptor-mediated endocytosis. It involves enriching the surface of nanoparticles with specific ligands, usually polypeptides or antibodies, that selectively bind to proteins overexpressed on cancer cells. Common targets for these guiding factors include receptors for transferrin, folic acid, or epidermal growth factor (EGF). Another group of compounds used to modify nanoparticles includes ligands with affinity for proteins overexpressed in the endothelial lining of tumour blood vessels, such as vascular endothelial growth factor (VEGF), αvβ3 integrins, vascular cell adhesion molecule-1 (VCAM-1), or matrix metalloproteases [39]. The functionality of surface modification of nanoparticles is discussed in Section 5 of the paper.

## 3. Methods of Synthesising Albumin Nanoparticles

**Figure 2:**
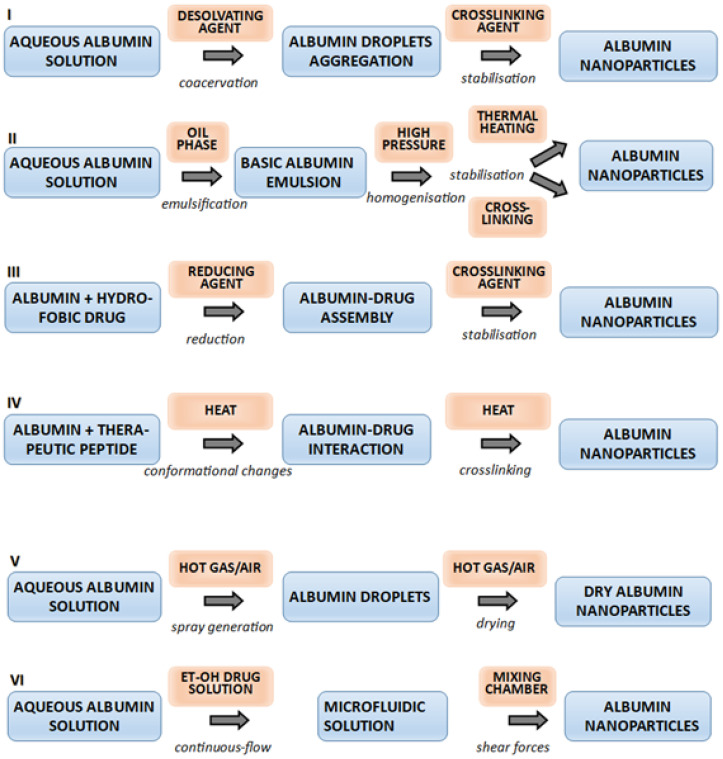
Popular methods for preparing albumin nanoparticles [28,42]: I—Desolvation; II—Emulsification; III—Self-assembly; IV—Thermal gelation; V—Nanospray drying; VI—Microfluidic technology.

Albumin nanoparticles can be synthesised using relatively simple chemical or physical methods without the need for specialised reaction conditions. The choice of technique depends on the physicochemical properties of the therapeutic component, the desired particle size, and the intended application. Human serum albumin (HSA) used in drug delivery systems (DDS) is obtained either directly from plasma by fractionation or through biotechnological methods employing genetically engineered *Pichia pastoris* yeast. In addition, albumin nanostructures can be fabricated from bovine serum albumin (BSA), rat serum albumin (RSA), or ovalbumin derived from hen egg white. Commonly applied techniques include desolvation, emulsification, nab technology, thermal gelation, spray drying, and ‘green chemistry’ approaches [5,7,40,41]. A schematic diagram of the steps involved in each preparation method is presented in Figure 2.

Desolvation involves continuously adding a dehydrating agent, such as ethanol, to an aqueous albumin solution maintained at a slightly acidic pH and room temperature with constant stirring. Under these conditions, albumin precipitates from the solution in the form of colloidal aggregates. These coacervates appear as droplets that contain significantly more colloidal particles than the surrounding solution. Next, glutaraldehyde is gradually added to induce crosslinking of the albumin, resulting in the formation of homogeneous, stable nanoparticles. Their size can be precisely controlled by adjusting parameters such as solution pH, the type of dehydrating agent, the final concentration of glutaraldehyde, temperature, and even mixing speed. This allows reproducible processing and the production of nanoparticles with stable, well-defined characteristics [43]. Importantly, the final product does not require additional purification, as the solvents used can be efficiently removed during lyophilisation, and the process can be easily up-scaled with a membrane contactor [44]. Optimisation studies have shown that the minimum concentration of glutaraldehyde required for crosslinking amino groups corresponds to 40% of all lysine residues in the albumin. It has also been demonstrated that temperature control alone can induce the polymerisation of albumin molecules without the need for a chemical crosslinker [45,46]. This is an advantageous phenomenon because some reports claimed the risk of toxicity caused by residual aldehydes in the glutaraldehyde-stabilised albumin nanoparticles [47]. Albumin crosslinking has likewise been achieved using physical methods, including ultraviolet or ionising radiation [48] or nontoxic, amine reactive crosslinkers like genipin or glucose [49]. The simplicity of the desolvation process facilitated its adaptation for large-scale nanoparticle production. Examples of technological improvements include the addition of ethanol through a semipermeable membrane (also known as a contactor) and the use of paddle stirrers combined with a high-pressure pump [50]. A more recent modification involving the sonication of an aqueous suspension of a drug and albumin before adding ethanol increased the drug encapsulation efficiency. Furthermore, replacing glutaraldehyde with N-(3-dimethylaminopropyl)-N′-ethylcarbodiimide enabled the fully controlled, simple, and reproducible synthesis of albumin nanoparticles with precisely defined sizes [51,52].

Emulsification, understood as encapsulation, is a technique in which an albumin solution is added to an oil phase containing the dissolved drug. Homogenisation of the mixture disperses the aqueous albumin suspension, leading to the formation of nanoparticles. These albumin nanoparticles are subsequently coagulated and stabilised through thermal denaturation or chemical crosslinking [53]. The critical limitation of this method is the need to use organic solvents to eliminate oil-phase residues and surfactants, which are frequently associated with adverse drug reactions. Additionally, it is challenging to obtain nanoparticles with diameters smaller than 500 nm by this technique [54].

Self-assembly is another method for producing albumin nanoparticles. The process is initiated by reducing the polarity of the albumin solution through the addition of a hydrophobic substance, and by decreasing the surface charge of the molecules through the blocking of free amino or carboxyl groups on amino acid side chains. Under these conditions, albumin molecules form microemulsions in the aqueous environment, generating vesicles with diameters up to 100 nm. The resulting polymeric albumin micelles can transport both hydrophobic and amphipathic drugs [55]. The challenges in producing albumin micelles include low storage stability, rapid disintegration in vivo, variable solubility, and the need to develop technology for large-scale production. An important application of the self-assembly approach is the development of cationic albumin nanoparticles as siRNA (small interfering RNA) carriers for the treatment of metastatic lung cancer. Cationic albumin molecules were produced by adding ethylenediamine, which formed stable amide bonds with free carboxyl groups. The modified albumin then spontaneously assembled into nanocomplexes with siRNA through electrostatic interactions with the negatively charged phosphate groups of ribonucleic acid. Cell line studies confirmed the efficiency of these nanoparticles in delivering specific siRNA and silencing the anti-apoptotic *Bcl-2* gene. Furthermore, in vivo studies in a B16 murine melanoma model demonstrated the ability of these nanoparticles to deliver siRNA to melanoma cells, resulting in reduced tumour mass and decreased lung metastases [56].

Thermal gelation and spray drying are less commonly used methods for producing albumin nanoparticles. The thermal gelation process is a multistep process, beginning with the mixing of an aqueous albumin solution with another protein of therapeutic potential. The mixture is then exposed to chemical and physical factors that induce protein unfolding, interaction, and nanoparticle formation, ultimately leading to nano-hydrogels. Nanostructures obtained by this method are typically spherical and exhibit a core–shell architecture. An example is a nanogel of spherical albumin nanoparticles with a lysozyme-filled core. The final product preserved its quality and long-term stability after lyophilisation [57].

Spray drying is a dehydration method used to convert colloidal suspensions into powder form. In this process, the liquid product is atomised using ultrasonic nozzles into a drying chamber, where it comes into contact with a stream of hot gas. This causes rapid solvent evaporation from the aerosol droplets, and the resulting powder particles are collected at the bottom of the chamber using electrostatic filters [58]. Modern spray dryers are suitable for producing large quantities of fine particles with continuous transitions through the successive stages of the process [59].

Microfluidic technology (MT) is an emerging method for nanoparticle preparation. The system uses a high-pressure delivery pump to generate tiny droplets by injecting the dispersed aqueous phase into an immiscible organic phase. Turbulence and laminar flow at the interface of the two phases create shear forces that induce mixing. Nanoparticle size can be precisely controlled by flow and shear forces, making microfluidics a stable, continuously operating, and easily monitored system for synthesising homogeneous protein nanoparticles. Moreover, the physicochemical properties of protein nanoparticles can be tuned by adjusting the alternating current electric field and flow rate [60]. Microfluidic technology has been applied to produce lipid, polymeric, inorganic, and protein nanoparticles, enabling high drug-loading efficiency, improved solubility, reduced cytotoxicity, and enhanced tumour cell internalisation [61,62]. More recently, a microfluidic co-flow method was developed to generate human serum albumin/celastrol nanoparticles. The resulting nanoparticles were size-homogeneous, stable, and crosslinked via naturally occurring cysteine residues formed during the assembly step. In addition to an encapsulation efficiency of approximately 75 ± 24%, this approach significantly improved the solubility of celastrol in the aqueous phase and reduced the cellular toxicity [63].

The Abraxis BioScience company developed a unique method for producing albumin-based nanoparticles, known as nab technology. The distinctiveness of this method lies in the fact that nanoparticles produced using nab technology do not require additional solvents or surfactants to be dispersed in aqueous solutions, making the formulation free of toxic additives. The process involves suspending a hydrophobic drug in an oil phase, mixing it with an aqueous albumin solution, and homogenising the mixture by forcing it through a narrow nozzle. Under elevated pressure, nanoparticles with diameters of 100–200 nm and high drug-loading capacity are formed. Subsequent steps include fluidisation, solvent evaporation, and filtration. Finally, the obtained emulsion can be lyophilised for long-term storage [64]. Table 1 summarises the main steps of nab technology, highlighting their role in nanoparticle production [15,65]. It should be emphasised that the first nanodrug approved by the FDA for the treatment of metastatic breast cancer, non-small cell lung cancer, and pancreatic cancer was manufactured using nab technology [25,66].

A separate area of interest is the formation of albumin nanocomplexes with radionuclide metals, as well as the synthesis of nanoparticles with an albumin coating (albumin corona). The most common examples are albumin complexes with radionuclides such as technetium-99m and indium-111, which are used for lung and gastrointestinal imaging, as well as for the sentinel lymph nodes location [67]. The synthesis of such nanoparticles is a multistep process that begins with the internal disulfide bonds reduction of albumin using mild reducing agents, such as glutathione. The aqueous suspension of albumin is then mixed with a suspension of the radionuclide in tert-butanol. As the solubility gradually decreases, co-precipitation of the protein and radionuclide occurs, followed by spontaneous complex formation after incubation at 37 °C. The most widely used preparations are Nanocoll, with an average albumin aggregate size of 8 nm, and Albures, with particle sizes ranging from 200 to 1000 nm [68].

## 4. Morphological Characterisation of Albumin Nanostructures with Their Potential Applications

Albumin nanostructures obtained using various techniques differ not only in size but also in shape and spatial organisation. The biophysical properties resulting from different morphologies of nanoparticles determine their use in multiple branches of medicine. A summary of the advantages and potential applications of the individual albumin nanoparticles is presented in Table 2.

## 5. Surface Modification of Nanoparticles

Albumin-based nanoparticles are being explored mainly as drug delivery systems for cancer treatment and diagnostic imaging, including liver or lung scans [82]. They are also being investigated for other medical applications, such as anti-inflammatory therapy, targeted delivery through the mucosa and blood–brain barrier, and addressing conditions like periodontal disease and neurological disorders [83,84]. The desirable features of albumin NPs for specific applications are often achieved through chemical modification or ligand incorporation on the nanocarrier surface.

Surface modification of nanoparticles is intended to enhance the therapeutic efficacy of drugs by prolonging their half-life. The albumin nanostructures in circulation can be coated with opsonins and cleared from the blood by the mononuclear phagocyte system [85]. This process can be slowed by modifying the nanoparticle surface with biologically inert polymers. Such modification also allows for the incorporation of targeting ligands that guide the drug to specific tissues [86,87]. Modifying agents can be conjugated directly to amino acid residues of albumin or attached via a biologically inert crosslinker. The compounds most commonly used for altering albumin nanoparticles include surfactants, polyethylene glycol (PEG), cationic polymers, folic acid salts, apolipoproteins, monoclonal antibodies, transferrin, and peptides [7,88,89].

Polymers used to stabilise albumin NPs and modulate drug release can be either chemically attached to the nanoparticle surface or conjugated with albumin before nanostructure synthesis. One example of a surface-active compound applied to enhance albumin nanoparticles is Polysorbate 80, a widely used emulsifier that stabilises aqueous suspensions of hydrophobic drugs. Coating doxorubicin-loaded albumin nanoparticles with Polysorbate 80 prolonged their half-life and reduced the toxicity to cardiac and testicular tissues in experimental animals. This protective effect was attributed to the slower metabolism of the nanoparticles by cells, which consequently reduced the intracellular cytostatic concentration in healthy tissues [90]. Polysorbate 80 has also been investigated for its ability to enhance nanoparticle penetration across the blood–brain barrier and to improve the bioavailability of orally administered antiviral drugs [91,92].

Coating nanoparticles with polyethylene glycol (PEG), a process known as PEGylation, has numerous beneficial effects, including reduced immunogenicity and prolonged biological half-life, which in turn enhances drug retention in the affected tissue. Another advantage is that the PEG forms a chemical barrier that slows drug diffusion from nanostructures, creating a depot-like system with sustained release [93,94]. PEG, combined with lactoferrin, has been used to modify the surface of bovine serum albumin (BSA) nanoparticles loaded with doxorubicin (DOX) to improve drug transport across the blood–brain barrier. The synthesis of this carrier involved a few steps: (i) formation of BSA–DOX nanoparticles via desolvation, (ii) conjugation of PEG2000 to the nanoparticle surface, and (iii) adsorption of lactoferrin through electrostatic interactions. Cytotoxicity assays against BCECs/C6 glioma cells, along with in vivo pharmacokinetic studies in rats bearing C6 tumours, demonstrated increased cytotoxicity toward tumour cells, prolonged blood half-life, and enhanced penetration into brain tissue compared with unmodified BSA–DOX nanoparticles [95].

Cationic polymers are employed in nanoparticle production to stabilise them and increase the resistance to proteolytic enzymes, resulting in a delay in drug release from nanostructures. The most commonly used are copolymers of lactic and glycolic acids, polycaprolactone, polyethyleneimine (PEI), and poly-L-lysine (PLL), as well as other thermosensitive polymers [16]. Modification of HSA–doxorubicin nanoparticles with PEI altered their surface charge, reduced opsonisation and improved system stability, leading to increased uptake by MCF-7 human breast cancer cells [88]. Similarly, PLL enhanced the stability of albumin nanostructures by increasing their resistance to proteolytic enzymes. BSA-siRNA nanocapsules in aqueous solutions were found to improve stability by adding both a higher molecular weight and a greater concentration of the PLL coating polymer [96].

Thermosensitive polymers are applied in the design of delivery systems with controlled drug release. One such system involves albumin nanoparticles conjugated with poly(N-isopropylacrylamide-co-acrylamide-co-allylamine) (PNIPAM–AAm–AA), developed for the controlled delivery of Adriamycin. Compared with unconjugated nanoparticles, these polymer-coated particles exhibited slower in vitro drug release in the presence of proteolytic enzymes, in a polymer mass-dependent manner. Moreover, they released the drug to HepG2 cells only at temperatures above the sol–gel phase transition [97]. These properties enable Adriamycin to be selectively targeted to tumour tissues in a temperature-dependent manner, exploiting the local hyperthermia characteristic of cancerous tissue.

Natural polysaccharide chitosan polymers are used to enhance the biocompatibility of drug delivery systems. The cationic nature of chitosan confers mucoadhesive properties, helping with the adhesion to cell membranes. Application of chitosan to the surface of insulin-loaded albumin nanostructures increased the intestinal absorption of the hormone and may potentially support the development of oral insulin formulations in the future [98].

Folate can serve as a targeting ligand due to its high affinity for folate receptors, which are overexpressed on the surface of many cancer cells. It is most commonly conjugated to proteins through a covalent bond between the carboxyl group of folic acid and the amino group of the protein [99]. Folate modifications have primarily been used in the design of cytostatic drug delivery systems and contrast agents for cancer imaging. An example is albumin nanospheres conjugated with folic acid, prepared by desolvation and developed as a platform for doxorubicin delivery to tumour tissue. These modified nanoparticles were efficiently internalised by HeLa cancer cells while showing no cytotoxicity towards normal aortic smooth muscle cells [100]. In other in vitro studies, BSA nanocapsules enriched with folate demonstrated increased transport and release of paclitaxel to prostate cancer cells compared to unmodified nanocapsules. Folic acid also improved the stability and solubility of these nanoparticles [101].

Another approach involved expanding the application of superparamagnetic iron oxide nanoparticles (γ-Fe_2_O_3_, SPIO NPs) by coating their surface with BSA and conjugating them with folic acid. In studies on the human glioma U251 cell line, this system showed high biocompatibility and effective cellular internalisation, allowing its use in MRI (magnetic resonance imaging)-based diagnostic imaging [102]. Moreover, incorporating folic acid into anticancer delivery systems may reduce the risk of tumour resistance to radiation and enable the application of highly potent cytostatics by targeted drug delivery [103,104].

Proteins with receptors abundantly expressed on the blood–brain barrier are commonly used to functionalise nanocapsule surfaces designed for central nervous system (CNS) delivery. These include transferrin, lactoferrin, and apolipoprotein A [105]. Human serum albumin (HSA) nanoparticles covalently bound to transferrin or to a monoclonal antibody against the transferrin receptor, and loaded with loperamide, an opioid that normally cannot cross the blood–brain barrier, demonstrated excellent CNS penetration, resulting in strong analgesic effects in an experimental animal study [106]. A similar outcome was observed for HSA nanocapsules containing loperamide and functionalised with apolipoproteins A-I, B-100, and E3 following intravenous administration [107]. Apolipoproteins are naturally recognised by endothelial cells of the blood–brain barrier. They are transported into the perineuronal space via receptor-mediated transcytosis, even when incorporated into larger complexes such as albumin nanocapsules. This process is mediated by membrane receptors of the LDLR family (low-density lipoprotein receptors) [108]. In addition to enhancing brain delivery, apolipoprotein modification prolongs the circulation half-life of the encapsulated drug and reduces its side effects [109].

Drugs can be directed to specific tissues by incorporating monoclonal antibodies (mAbs) into delivery systems. The primary function of such modified NPs is to achieve targeted cytostatic transport by exploiting the overexpression of specific antigens on malignant cells. The most commonly used monoclonal antibodies for functionalising nanostructures include Trastuzumab (used in breast cancer), Cetuximab (used in head, neck, kidney, and other cancers), and Abituzumab (used in prostate and colon cancer) [110]. Herceptin (Trastuzumab) is a humanised antibody against HER2 (human epidermal growth factor receptor 2), an antigen overexpressed on certain breast cancers. Biotinylated Herceptin was conjugated with avidin-modified albumin nanoparticles to deliver antisense oligonucleotides to BT-474 cells. This strategy successfully silenced the Plk-1 proto-oncogene and reduced the synthesis of the encoded protein [111]. Comparable results were obtained with Cetuximab and Abituzumab, monoclonal antibodies targeting the epidermal growth factor receptor (EGFR) and the αV integrin subunit, respectively, when used to functionalise albumin-based carriers for drugs studied in colon cancer and melanoma models [112,113].

Other proteins and peptides used to modify albumin nanostructures include TRAIL (tumour necrosis factor-related apoptosis-inducing ligand), a ligand for death receptors that induces pro-apoptotic signalling in cancer cells; the RGD peptide, a cyclic tripeptide (Arg-Gly-Asp) with high affinity for integrin αvβ3, which is overexpressed in pancreatic adenocarcinoma vasculature; and TAT (HIV-1 transcription factor)-penetrating peptides. TRAIL has been employed to create a synergistic system with doxorubicin (DOX) to enhance the anticancer efficacy of albumin nanoparticles produced using nab technology. TRAIL-loaded nanoparticles exhibited stronger pro-apoptotic effects on colon cancer cell lines than those containing DOX alone and more effectively inhibited tumour growth in HCT116 xenograft mice, demonstrating a promising approach to treating lower gastrointestinal cancers [114]. Cyclic RGD (cRGD) peptides are conjugated to albumin molecules using chemical crosslinkers to create drug delivery systems that target integrin αvβ3 receptors on cancer cells. Examples of crosslinkers include maleimide and carbodiimide chemistry, used to form stable bonds between the peptide and albumin [16]. HSA nanoparticles loaded with Gemcitabine for targeted cancer therapy were synthesised using sulfosuccinimidyl-(4-*N*-maleimidomethyl)cyclohexane-1-carboxylate (Sulfo-SMCC) crosslinkers and formulated based on the nanoparticle albumin-bound (nab) technology. In vitro experiments on BxPC-3 cell lines confirmed increased uptake of cRGD-functionalised nanoparticles, suggesting strong potential for in vivo applications [115]. The TAT peptide improves transfection efficiency by enhancing the permeability of cell membranes to nucleic acids [116]. An application of TAT in albumin nanoparticles involved plasmid DNA delivery systems. When comparing cRGD- and TAT-functionalised nanoparticles, the TAT-modified carriers demonstrated superior efficiency in transfecting HEK293T cells in vitro [117]. Such nanostructures may represent safe and effective carriers for genetic material in future gene therapy applications.

## 6. Development and Future of Albumin-Based Nanosystems

Currently, most research is focused on the synthesis of advanced diagnostic–therapeutic platforms that combine both a tumour-imaging component and a therapeutic agent, such as a light-activated compound. This integration of therapy with diagnostics and treatment monitoring is referred to as theranostics [118,119]. Theranostic nanoparticles are typically constructed as isotope-enriched aggregates of cytostatics with a carrier. Imaging techniques such as MRI enable monitoring of their activity in the human body. Tracking the in vivo distribution of theranostic nanoparticles makes it possible to verify whether the drug has been internalised by cancer cells. Thus, the design of such multi-component theranostic systems aims not only to achieve targeted treatment but also to enable image-guided therapy. One approach involves albumin nanocomplexes incorporating photosensitising compounds and near-infrared (NIR)-absorbing dyes to create a system capable of resonance energy transfer via the Förster mechanism. In the excited state, energy is transferred from the photosensitiser to the dye. Upon NIR illumination, the dye releases this absorbed energy back to the photosensitiser, inducing fluorescence and generating reactive oxygen species. This dual functionality enables real-time fluorescence tracking of drug distribution in the body, combined with spatially controlled activation of the drug by infrared light once it reaches the tumour tissue. Such targeted activation in photodynamic therapy can effectively inhibit tumour growth while minimising adverse skin reactions frequently associated with conventional phototherapy [120]. Another promising design includes gold–albumin aggregates in the form of nanoclusters, which can be formulated into nanoparticles loaded with cytostatic agents such as doxorubicin (DOX). Nanoparticles prepared in this way (Au–BSA–DOX) and tested on HeLa cervical cancer cells were efficiently internalised, with the drug reaching the cell nucleus, as confirmed by two-photon confocal microscopy. Luminescence from the gold–albumin aggregates enabled nanoparticle tracking, while DOX fluorescence allowed monitoring of drug release within cells. Significantly, the excitation and emission wavelengths of these nanoparticles fell within the near-infrared (NIR) range (650–900 nm), which is safe for humans [121]. In other studies, gold–albumin nanoparticle aggregates were conjugated with a cisplatin derivative and folic acid to create an integrated theranostic system for targeted diagnosis and chemotherapy of breast cancer. This folic acid conjugation significantly enhanced the intracellular drug transport and cytotoxicity against 4T1 cells, and in mice bearing breast tumours, the NPs inhibited both tumour growth and the formation of lung metastases. Importantly, these nanoparticles could also be monitored non-invasively in vivo using NIR imaging [122].

Theranostic properties can be acquired through surface modification for albumin nanocomplexes with inorganic molecules lacking intrinsic fluorescence. For example, the aggregation of ferromagnetic gadolinium (Gd) with albumin resulted in nanoparticles suitable for MRI-based tumour imaging [123]. Additional surface modification with cyanine dyes provides photothermal and photoacoustic functionality upon irradiation. Such nanoparticles demonstrated increased accumulation in the tumour and induced cancer tissue reduction by photothermal effects. Moreover, the presence of Gd ions enabled combined photodynamic therapy with simultaneous in vivo nanoparticle monitoring (MRI/NIRF) [124].

Recently, several studies have been undertaken to synthesise metal aggregates with photosensitising properties using an albumin matrix for photodynamic cancer therapy. Research involved various metal-based nanoparticles and nanoclusters (e.g., gold, palladium, copper, ruthenium, iridium complexes) that can act as photosensitisers or enhance the photodynamic effect. Upon light activation, these materials generate reactive oxygen species that damage and kill cancer cells [125]. The albumin-templated nanoclusters can be ultrasmall, as in the case of Ag nanoclusters (<2 nm), with strong singlet oxygen generation capacity for photodynamic therapy (PDT). Such albumin-based nanostructures containing silver ions generated substantial amounts of singlet oxygen upon irradiation, and due to intrinsic fluorescence, enabled in vivo monitoring of drug accumulation in tumour tissue [126]. The most promising are albumin nanoparticles designed for combination therapy, in which a chemotherapeutic agent is co-loaded with a ‘trigger’ factor that enables drug release only at a specific site or under particular conditions, such as altered pH, redox status, or light exposure [127].

## 7. Outlook

The use of albumin-based therapeutic nanosystems in medicine offers desirable biocompatibility and enhanced drug efficacy, not only in oncology but also in rheumatology and neurology [128,129,130,131]. Studies on albumin-based formulations have revealed that such approaches may be particularly effective for drugs with poor pharmacokinetics, limited efficacy, or high toxicity. Albumin-based nanoparticles exploit receptor-mediated cellular uptake, thereby minimising side effects on healthy tissues. Moreover, binding to albumin significantly enhances drug accumulation in cancerous or inflamed tissues [132,133]. Encapsulation within albumin nanoparticles increases stability, protects against enzymatic degradation, and improves penetration into the affected tissue [134,135]. While most albumin-based preparations remain in preclinical or clinical evaluation, several have already received approval for therapeutic use (e.g., Abraxane^®^, Fyarro^®^) or for imaging applications (e.g., IC-GREEN, Pulmotech MAA, MEGATOPE, Nanocoll) [136,137].

This review highlights that albumin NPs offer a promising and versatile platform for addressing the issues of targeted drug delivery, although some hindrances must be overcome before they can be widely used in medicine. Limitations of albumin NPs include challenges in large-scale production, batch-to-batch variability, potential toxicity from crosslinking agents, unsatisfactory blood–brain barrier penetration, nonspecific uptake by the immune system, and complex synthesis methods requiring organic solvents or cationic polymers. Even a slight change in synthesis conditions can affect size, drug loading capacity, and incorporation of functionalised ligands, making the production of consistent and high-purity NPs difficult. Although many studies report no acute toxicity, chronic exposure to nanoparticles modified with large peptides or proteins could trigger an immune response. The considerable production costs resulting from quality control requirements and the biological safety of the product must also be taken into account when developing a new albumin-based transport system for drug targeting [138]. In conclusion, the future of targeted therapy may heavily rely on albumin-based nanosystems, bearing in mind that the translation bench-to-bedside is contingent upon addressing several significant limitations and challenges.

## Figures and Tables

**Table 1:** Nab technology stages for albumin nanoparticle preparation.

Step	Technique	Effect
Crude emulsification	Mixing hydrophobic drug with albumin under low shear forces	Formation of a crude emulsion
Formation of Nanoparticles	High-pressure homogenisation/emulsification	Self-assembly of albumin into nanoparticles with the drug bound via intramolecular crosslinking
Stabilisation and Purification	Solvent evaporation, filtration, lyophilisation	Stable nanoparticles of desired size; removal of free drug and unbound albumin

**Table 2:** Morphology, synthesis techniques, advantages, and potential applications of albumin nanostructures.

Nanostructure Morphology	Technique	Advantages	Potential Application	Ref.
Spherical nanoparticles	nab technology; nanospray drying	Easy and reproducible synthesis allowing surface modification and enrichment; scalable process with controllable particle size	Carriers for poorly soluble anticancer drugs and gene therapy	[69,70,71]
Microspheres	Stabilisation by crosslinking and high temperature	Improved pharmacokinetic properties and prolonged circulation time	Targeted therapy of solid tumours	[72,73]
Microbubbles	Dispersion of albumin solution with inert gas by sonication	Automated and easily controlled synthesis	Ultrasound-triggered carriers for contrast agents and gene delivery	[74,75]
Nanocapsules	Micronebulisation of an aqueous mixture of albumin and drug into an oil phase	Simple solvent-evaporation procedure; easy to set on an industrial scale	Anti-inflammatory and peptide drug carriers	[76,77]
Albumin-coated liposomes	Electrostatic interactions between albumin and cationic liposomes	High encapsulation efficiency; enhanced cellular uptake	Carriers of antisense oligonucleotides; transepithelial transport	[78,79]
Magnetic Fe_3_O_4_ nanoparticles coated with albumin	Coprecipitation	Stabilisation and enhanced delivery to cancer cells	Targeted cancer therapy via magnetic hyperthermia; contrast agents	[80,81]

## Data Availability

No new data were created or analysed in this study. Data sharing is not applicable to this article.

## Abbreviations

The following abbreviations are used in this manuscript:

DDS	Drug Delivery System
FDA	Food and Drug Administration
BSA	Bovine Serum Albumin
RSA	Rat Serum Albumin
PEG	Polyethylene Glycol
DOX	Doxorubicin
PEI	Polyethyleneimine
PLL	Poly-L-lysine
siRNA	Small Interfering RNA
CNS	Central Nervous System
EGFR	Epidermal Growth Factor Receptor
TRAIL	TNF-Related Apoptosis-Inducing Ligand
TAT	HIV-1 Transcription Factor
EPR	Enhanced Permeability and Retention
SPARC	Secreted Protein, Acidic and Rich in Cysteine
HER2	Human Epidermal Growth Factor Receptor 2
NIR	Near-Infrared
MRI	Magnetic Resonance Imaging
